# Spontaneous pneumothorax as a first sign of pulmonary carcinoma

**DOI:** 10.1186/1477-7819-7-57

**Published:** 2009-06-30

**Authors:** Vladislavas Vencevičius, Saulius Cicėnas

**Affiliations:** 1 Department of Thoracic Surgery and Oncology, Institute of Oncology, Vilnius University, Santariškių 1, Vilnius, Lithuania; 2 Vilnius University, Medical Faculty, Institute of Rehabilitation, Sport Medicine and Nursing, Vilnius, Lithuania

## Abstract

**Background:**

Spontaneous pneumothorax (SP) is a rare manifestation of lung cancer. The mechanisms by which pneumothorax occurs in lung cancer is not clear, resulting in different views being expressed.

**Case presentation:**

Here we present a case in which pneumothorax occurred as a first manifestation of lung cancer. The chest x-ray of a 68 year old man revealed a right partial pneumothorax. VATS was then performed: the visceral pleura lying over segment S_3 _was destroyed and air leaks were found in this section. Pathologic examination of the biopsy specimen revealed non-small cell carcinoma. Thoracoscopic talc pleurodesis was performed.

**Conclusion:**

Spontaneous pneumothorax in association with lung cancer is rarely seen. Pneumothorax can be the first sign of lung cancer. The most common possibility for SP complicating lung cancer is the tumor necrosis mechanism or, in separate cases, rupture of the emphysematous bullae. Lung cancer should always be considered as a possible cause of SP in elderly patients or in heavy smokers.

## Background

SP is generally attributed to a rupture of the sub-pleural blebs or emphysematous bullae [1]. This can complicate primary or secondary lung tumors. SP in primary pulmonary neoplasm or lung metastasis is very rare and the estimated rate of joint occurrence is approximated to be between 0,03 and 0,05 percent for primary lung cancer [1-5]. Pneumothorax due to primary lung cancer is also rare and prognosis is poor because most often the cancer is either at an advanced stage or the diagnosis of cancer was delayed [1,5].

## Case presentation

**Figure 1:**
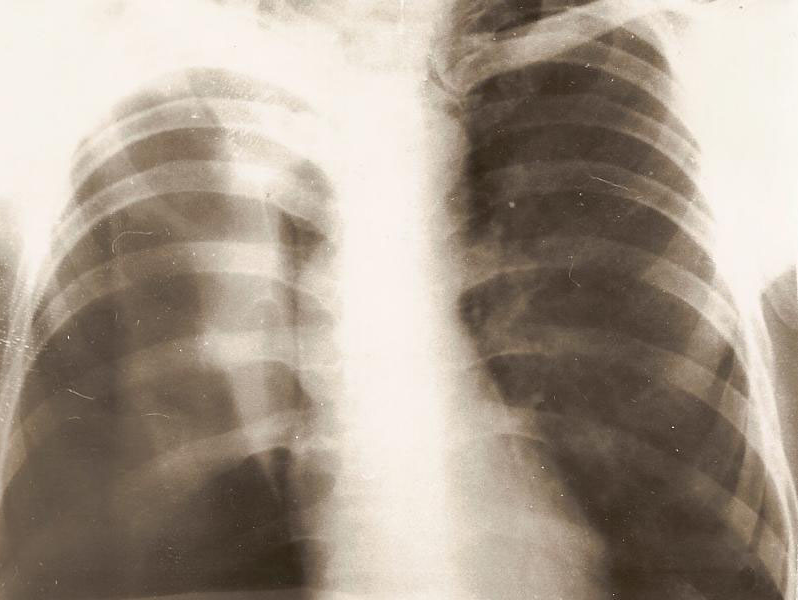
**Right spontaneous pneumothorax VATS: right S_3 _segment granulations in centrally visceral pleura defect**. Biopsy: squamous cell cancer.

**Figure 2:**
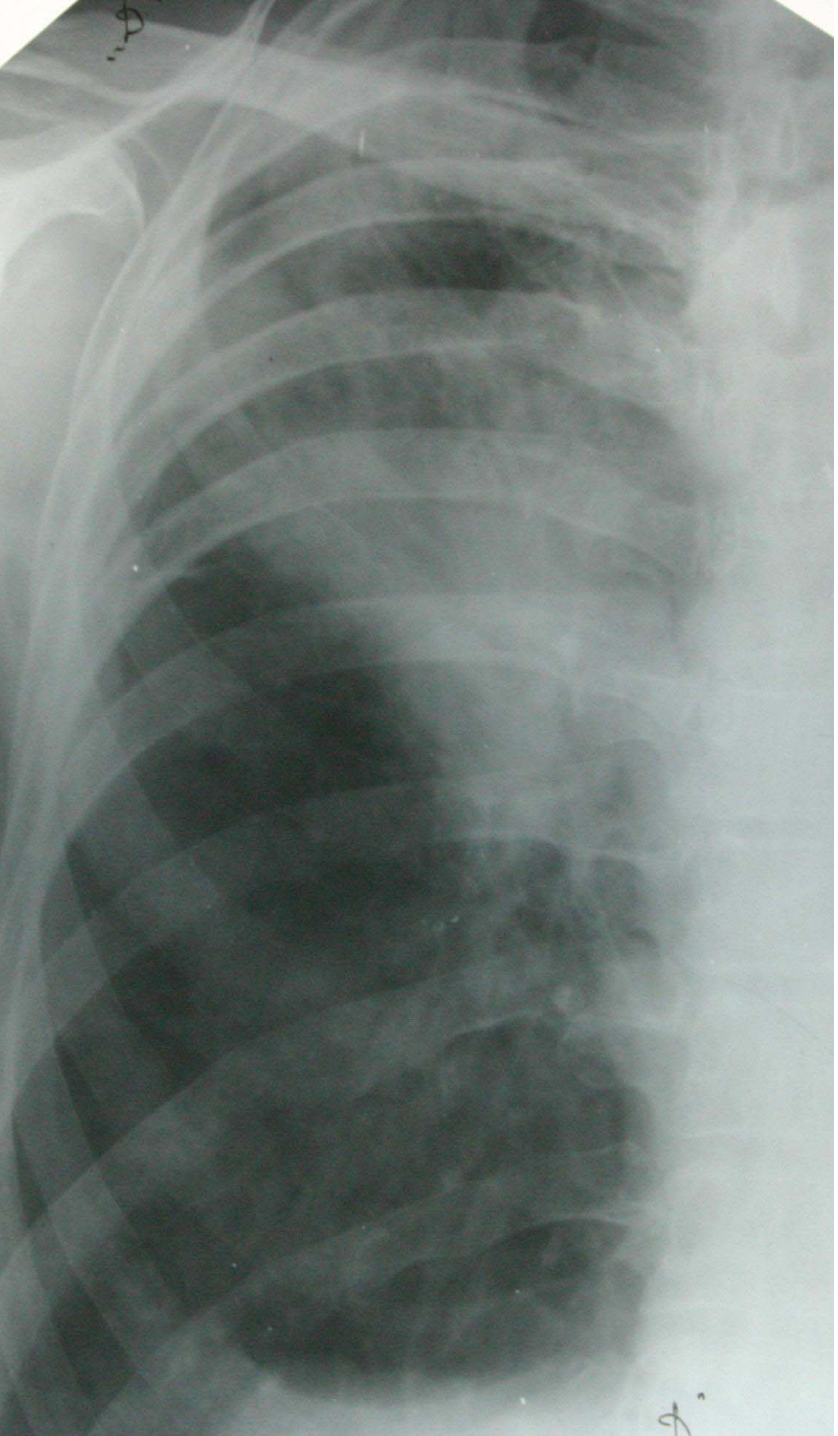
**Picture shows: after active pleural drainage – lung expended**. Right upper lobe: non homogenic infiltration – tumour.

A 68 year old man was admitted in September 2007 with complaints of chest pain, dyspnea and cough in the past 6 days. He was a heavy smoker. Physical examination revealed tachycardia and tympanic percussion over the right chest, dullness with decreased breath sounds over the right lower chest. His chest radiograph of the thorax showed a lesion in the upper zone of the right lung and partial pneumothorax (Figures 1, 2).

Routine investigations revealed Hb: 8%, TLC: 9700/cumm, DLC: P62 L 38 cumin and ERS 20 mm in the first hour. Blood urea and sugar, etc. were normal. Direct smear examination of the sputum was negative for acid fast bacilli as well as malignant cells.

**Figure 3:**
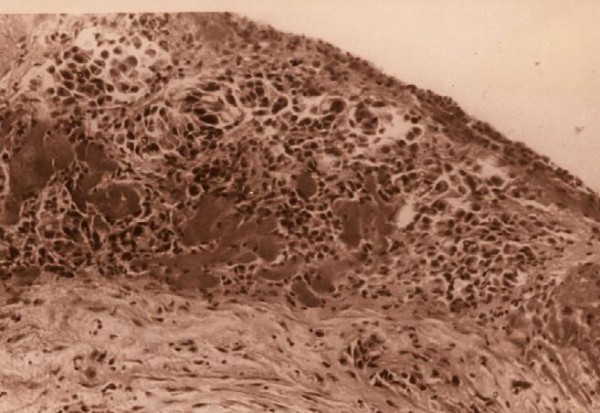
**Microview of biopsy specimen H & E stain, × 20 Tumor cells**.

Primary lung carcinoma was suspected. Thorascopy (VATS) was performed: the visceral pleura lying over segment S_3 _was destroyed and air leaks were found in this section. Histological examination of the biopsy specimen (S_3_) revealed non-small cell cancer (Figure 3). When bronchoscopy was performed, compression invasion of the right upper lobe bronchus was noted, but histological examination of the bronchoscopic biopsy specimen was negative (T2 b N0 M 0 stage II A).

Thoracoscopic talc pleurodesis was performed. Chest drains were removed after 6 days. Because the patient refused surgery, he then received chemotherapy.

## Discussion

Spontaneous pneumothorax is divided into primary and secondary. Primary SP most commonly afflicts the young and healthy. The secondary type can develop with obstruction, infection, infarction, neoplasm and diffuse lung disease.

SP as a complication of primary lung carcinoma (LC) is rare [6,7]. It is estimated that only 2% of all SP is coexistent with malignant lung diseases, either primary or secondary. This tumor complication must be especially considered in older patients [8].

To date, among the 1200 adults who were found to have SP from 1970–2007, 37 (3%) had lung cancer. In all such patients, the pneumothorax occurred in the same side as the carcinoma. The main cause of SP was the rupture of a necrotic tumor nodule or necrosis of subpleural metastases (for 21 patients). It also became the communication cause between the bronchus and pleural cavity, producing a bronchopleural fistula that resulted in pneumothorax. We demonstrate that these case reports of lung cancer with pneumothorax are a rare complication of primary lung carcinoma.

The mechanism producing pneumothorax from lung cancer is not well understood, but a number of theories have been advanced. The first is that it may be the result of tumor necrosis – rupture of the necrotic neoplastic tissue in the pleural cavity [9]; the second, that it may be caused by the rupture of the necrotic tumor nodule or necrosis of subpleural metastases [5]. A third is cancer of the check valve mechanism: the tumor at the lung periphery can obstruct bronchioles and lead to local overdistention and rupture of the lung [10]. The fourth is that most patients with lung cancer have chronic bronchitis or emphysema bullae and these bullae may rupture following the disturbance of the lung architecture due to bronchial cancer [11].

Pneumothorax related to therapy has been reported in patients receiving chemotherapy and/or radiotherapy for lung cancer [12]. There is the possibility that SP and lung cancer are two independent and incidental processes. These theories suggest that lung cancer should always be considered as a possible cause of SP in older patients [13].

## Conclusion

Spontaneous pneumothorax in association with lung cancer is rarely seen. Pneumothorax can be the first sign of lung cancer. The most common possibility for SP complicating lung cancer is the tumour necrosis mechanism or, in separate cases, rupture of the emphysematous bullae. Lung cancer should always be considered as a possible cause of SP in elderly patients or in heavy smokers.

## Consent

Written informed consent was obtained from the patient for publication of this case presentation and accompanying images. A copy of the written consent is available for review by the Editor-in-Chief of this journal.

## Competing interests

The authors declare that they have no competing interests.

## Authors' contributions

VV wrote the manuscript, sent the specimen to the pathologist, prepared the material for publication and operated the patients. SC collected data on a number of patients, operated on them, treated them and used diagnosis methods.
